# Oxidative Stress in Parkinson's Disease: A Systematic Review and Meta-Analysis

**DOI:** 10.3389/fnmol.2018.00236

**Published:** 2018-07-05

**Authors:** Zexu Wei, Xiaowan Li, Xixi Li, Qingshan Liu, Yong Cheng

**Affiliations:** Key Laboratory of Ethnomedicine for Ministry of Education, Center on Translational Neuroscience, College of Life and Environmental Sciences, Minzu University of China, Beijing, China

**Keywords:** oxidative stress, Parkinson's disease, peripheral blood, cerebrospinal fluid, meta-analysis

## Abstract

Oxidative stress has been suggested to play a key role in Parkinson's disease, but inconsistent results were found in clinical studies. This study sought to quantitatively summarize the blood and cerebrospinal fluid (CSF) oxidative stress marker data in PD patients. We performed a systematic search of PubMed and Web of Science, and studies were included if they provided data on peripheral blood and CSF oxidative stress marker concentrations in PD patients and healthy control (HC) subjects. Data were extracted by three independent investigators from 80 included studies encompassing 7,212 PD patients and 6,037 HC subjects. Of the 22 oxidative stress markers analyzed, random effects meta-analysis showed that blood concentrations of 8-OhdG, MDA, nitrite, and ferritin were increased in patients with PD compared with HC subjects. In contrast, we showed that blood levels of catalase, uric acid, glutathione, and total-cholesterol were significantly down-regulated in patients with PD when compared with controls. There were no significant differences between PD patients and HC subjects for blood, Mn, Cu, Zn, Fe, SOD, albumin, glutathione peroxidase, vitamin E, ceruloplasmin, triglycerides, lactoferrin, transferrin, LDL-cholesterol, and HDL-cholesterol. Due to the limited number of CSF studies with small sample size, this meta-analysis only showed non-significant association between CSF 8-OhdG and PD. The findings of our meta-analysis demonstrated higher blood concentrations of 8-OhdG, MDA, nitrite and ferritin, and lower blood concentrations of catalase, uric acid, glutathione and total-cholesterol in PD patients, strengthening the clinical evidence that PD is accompanied by increased oxidative stress.

## Introduction

Parkinson's disease (PD) is the second most common neurodegenerative disease after Alzheimer's disease (Bhimani, 2014). Clinically, this disorder has been mainly characterized by four motor-related symptoms: resting tremor, rigidity, bradykinesia, and postural instability (Jankovic, 2008), although the importance of non-motor features in PD such as depression and sleep disorders have been more recognized in recent years (Maass and Reichmann, 2013). Pathologically, PD is characterized by the progressive loss of dopaminergic neurons in the substantial nigra pars compacta, which is considered to be responsible for the motor symptoms of the disease (Jiang and Dickson, 2018). Additionally, proteinaceous inclusions known as Lewy bodies found in various brain regions of PD patients, have been demonstrated to occur before the loss of dopaminergic neurons (Braak et al., 2003). Although the mechanisms underlying the pathophysiology of PD have been far from fully understood, increasing evidence suggest that inflammation and oxidative stress play critical roles in the cascade of events leading to the degeneration of dopaminergic neurons (Gaki and Papavassiliou, 2014; Stojkovska et al., 2015). The involvement of inflammatory response in PD is supported the activation of microglial cells and reactive astrogliosis in the brains of patients with PD (Ouchi et al., 2005; Niranjan, 2014).

Oxidative stress reflects an imbalance between the production of free radicals and the body's ability to detoxify the toxic effects through antioxidants. Free radicals that mostly damaging to cells include hydrogen peroxide, the hydroxyl radical, nitric oxide (NO), and the superoxide radical, whereas the important antioxidants in human body include SOD, catalase, glutathione, and uric acid. SOD catalyzes the conversion of superoxide radicals to hydrogen peroxide, and then hydrogen peroxide is converted to water and oxygen by catalase and glutathione peroxidase. Glutathione peroxidase also converts nitrate to nitrite, which indicates the NO activity. Uric acid is converted by xanthine oxidase during the rate-limiting step of purine catabolism. In addition, 8-OhdG is an important marker for DNA damage, and malondialdehyde (MDA) is an important end product for lipid peroxidation. The failure of antioxidant defense system to protect against free-radical generation damages all components of the cell, including DNA, lipids and proteins, eventually leading to cell death, which has been considered to be involved in the development of PD (Dias et al., 2013; Hauser and Hastings, 2013).

In addition, efforts have been made to identify biomarkers reflective of inflammation and oxidative stress in PD, this is of potential importance not only to increase our knowledge of PD onset and development but also to support the diagnosis of PD and to monitor PD motor and non-motor progression. These led to a large number of studies to analyze levels of inflammatory cytokines and oxidative stress markers in circulation of PD patients. Although clinical data were inconsistent for individual biomarkers and across studies, a meta-analysis included 25 studies with 2654 individuals demonstrated that PD patients had elevated levels of blood inflammatory cytokine interleukin-6 (IL-6), tumor necrosis factor-α (TNF-α), IL-1β, IL-2, IL-10, C-reactive protein (CRP) and regulated on activation, normal T cell expressed and secreted (RANTES) compared with healthy control (HC) subjects, thus clarifying the peripheral blood inflammatory cytokine profile in patients with PD (Qin et al., 2016b). More clinical studies showed increased levels of circulating oxidative stress markers and decreased levels of antioxidants in PD patients, especially in peripheral blood (Kalra et al., 1992; Alimonti et al., 2007; Chen et al., 2009; Vinish et al., 2011; Medeiros et al., 2016; Yuan et al., 2016). However, significant heterogeneity among studies and negative results have been observed for the imbalance of oxidative stress pathway. Therefore, a systematic review with meta-analysis is needed to address the inconsistent clinical data for the oxidative stress markers (oxidative stress products and antioxidants) in PD.

In this study, we undertook the first systematic review with meta-analysis of studies measuring cerebrospinal fluid and peripheral blood levels of oxidative stress products and antioxidants in patients with PD compared with HC subjects, and we also used meta-regression and sub-group analyses to adjust variables that may explain the heterogeneity among studies.

## Methods

The meta-analyses implemented in this study conform to the instructions that are recommended by the PRISMA statement (Preferred Reporting Items for Systematic Reviews and Meta-analysis; Moher et al., 2009).

### Search strategy and study selection

A systematic review of peer-reviewed English articles from the databases of PubMed and Web of Science were performed by three investigators from September 2017 to March 2018. The search term included in the databases was: Parkinson's disease and (oxidative stress or superoxide dismutase or malondialdehyde or glutathione or catalase or hydroxyguanosine or uric acid or ceruloplasmin or transferrin or copper or low density lipoprotein or cholesterol). Original clinical studies that reported data on the concentrations of oxidative stress markers in patients with PD and healthy controls (HC) were included. Exclusion criteria were (1) no necessary data; (2) oxidative stress markers were measured in animal models; (3) no HC subjects; (4) samples overlapping with other studies; (5) *in vitro* data; (6) blood samples were taken before patients diagnosed as PD; (7) suffering from serious complications; (8) samples derived from postmortem brain; (9) individual markers were studied in less than 3 articles.

### Data extraction

One investigator extracted the data from the included articles in the meta-analysis, and the data was verified by another investigator. The primary outcomes extracted were oxidative stress marker concentrations, sample size and standard deviation to generate effective size (ES). Data on age, gender, country, publication year, PD duration, L-dopa duration, mean H and Y scale, mean UPDRS, BMI, sampling source, diagnosis, assay type, medication, and comorbidity were also extracted for potential moderator analysis (eTable).

### Statistical analysis

All statistical analyses were performed by the Comprehensive Meta-analysis Version 2 software. The ES was mainly generated by sample size, mean concentration and standard deviation (SD), or sample size and *P* value if the data for mean concentration and SD were not available. The standardized mean difference of oxidative stress marker concentration between PD patients and HC subjects was calculated as ES, and converted into Hedge's g statistic, which provides an unbiased adjusted ES for sample size (Qin et al., 2017a). We calculated an ES estimate for each oxidative stress biomarker assessed in the studies included in the meta-analysis. A random effects model was chosen for the meta-analysis because it is a more conservative model, as it produces a wider 95% confidence interval than the fixed effect model (sample size determines the study weight) if the between-study heterogeneity is significant (Qin et al., 2016a). The robustness of the outcome of the meta-analysis was assessed by sensitivity analysis, which was undertaken by removing one study at a time.

Between-study heterogeneity was assessed by Cochrane *Q* test and *I*^2^ statistic (Qin et al., 2017b). *P* < 0.10 was considered statistically significant for the Cochrane *Q* test. The inconsistency across studies was decided by *I*^2^ index to evaluate the impact of heterogeneity, *I*^2^ of 0.25, 0.5, 0.75 indicate small, moderate, high levels of between-study heterogeneity, respectively. To address the between-study heterogeneity, subgroup analyses and unrestricted maximum-likelihood random-effects meta-regressions of ES were performed to analyze whether the categorical and continuous variables affected the outcomes of the meta-analysis. Potential publication bias was determined by Egger's test, which assessed the asymmetry of funnel plot.

We set the statistical significance at *P* < 0.05 except where noted, *p* < 0.1 is considered as a trend.

## Results

**Figure 1:**
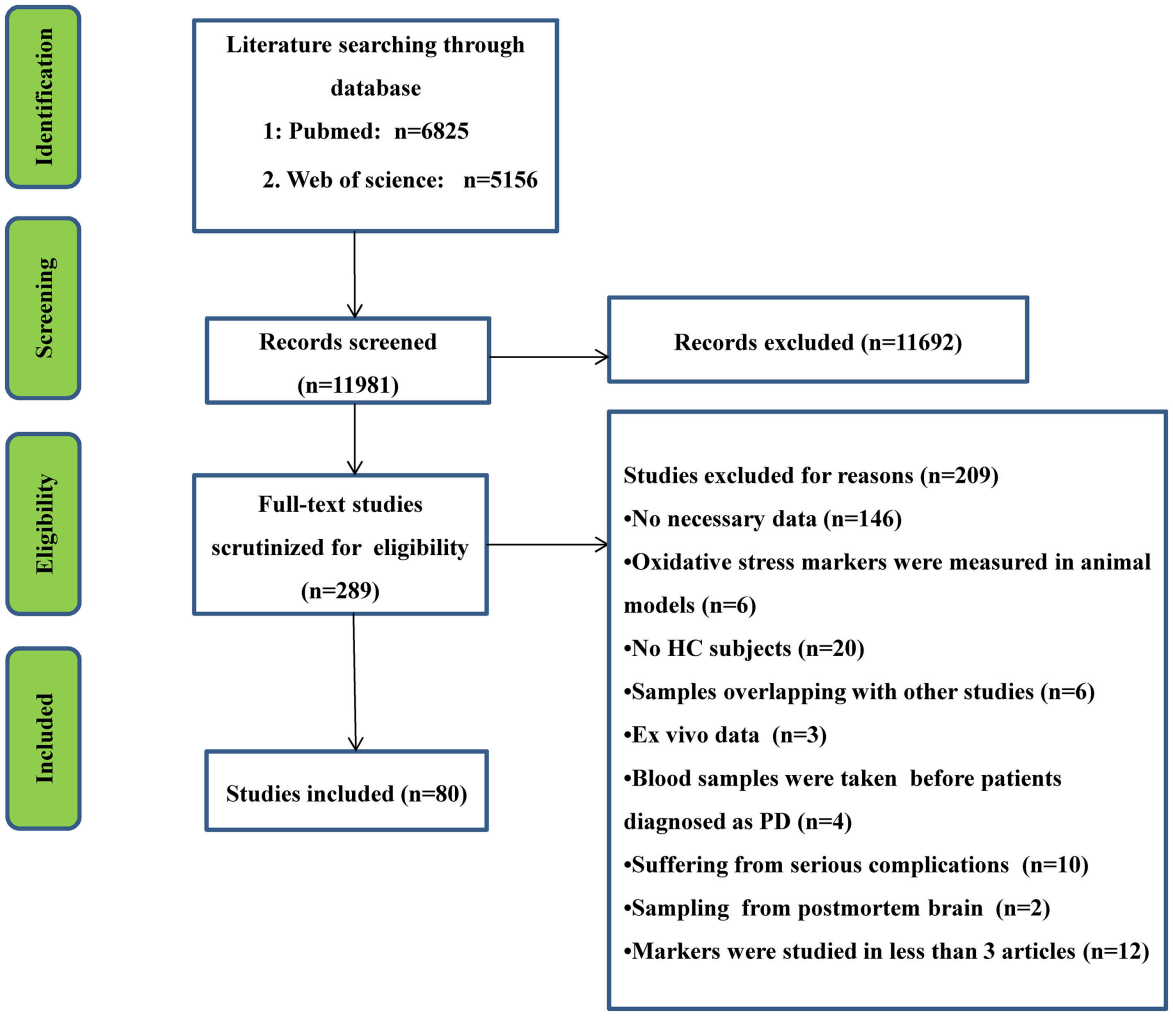
PRISMA flowchart of the literature search.

The initial search generated 6,825 records from PubMed database and 5,156 records from Web of Science, and then the titles and abstracts were screened. After the screening, 289 articles related to the present study were selected for full text scrutiny. Some studies were excluded because they lacked the necessary data (146 studies); oxidative stress markers were measured in animal models (*n* = 6); lack of a HC group (20 studies); had samples that overlapped with other studies (6 studies); *in vitro* data (3 studies); blood samples were taken before the patients were diagnosed as PD (4 studies); PD patients suffered from complications (10 studies); samples derived from postmortem brain (2 studies); individual makers were studied in less than 3 articles (12 studies). Therefore, a total of 80 studies including 13,249 unique study participants, with 7,212 PD patients and 6,037 HC subjects were included in the meta-analysis. A flow chart summarizing the study selection process was presented in Figure 1, the 80 included studies were provided as eReference.

### Association of PD with blood oxidative stress markers

Random-effects meta-analysis showed that patients with PD had significantly higher levels of blood oxidative stress markers compared with HC subjects for ferritin, 8-OhdG, nitrite, and MDA. In contrast, concentrations of uric acid, catalase, glutathione, and total-cholesterol were significantly lower in PD patients compared with HC subjects (Table 1, eFigures 1–3). Furthermore, blood levels of Mn, Cu, Zn, Fe, SOD, albumin, glutathione peroxidase, vitamin E, ceruloplasmin, triglycerides, LDL-cholesterol, lactoferrin, transferrin, and HDL-cholesterol did not show significant differences between PD patients and HC subjects (Table 1).

**Table 1:** Summary of Comparative Outcomes for Measurements of Blood Marker Levels.

**Cytokine**	**No. of studies**	**No. with PD/controls**	**Main effect**			**Heterogeneity**				**Publication bias**	
			**Hedges *g* (95% CI)**	***z* score**	***p* value**	***Q* statistic**	***df***	***p* value**	***I*^2^ statistic**	**Egger intercept**	***p* value**
Ferritin	8	635/444	0.659 (0.055 to 1.264)	2.138	0.033	142.439	8	< 0.001	94.384	6.58410	0.11877
Uric acid	18	2,818/2,588	−0.500 (−0.649 to −0.350)	−6.563	< 0.001	97.551	17	< 0.001	82.573	2.02674	0.08666
8-OHdG	4	349/197	0.772 (0.105 to 1.439)	2.268	0.023	27.730	3	< 0.001	89.181	−5.64137	0.18118
MDA/TBARS	10	664/641	0.836 (0.367 to 1.305)	3.492	< 0.001	124.798	9	< 0.001	92.788	−1.96281	0.58090
Nitrite	6	259/282	0.604 (0.085 to 1.124)	2.279	0.023	36.220	5	< 0.001	86.195	1.67843	0.61417
Catalase	6	203/176	−0.548(−1.051 to −0.044)	−2.133	0.033	26.366	5	< 0.001	81.036	0.87309	0.82923
Mn	7	497/514	0.037(−0.404 to 0.478)	0.165	0.869	74.685	7	< 0.001	90.627	2.43152	0.50465
Cu	13	831/905	0.354 (−0.256 to 0.964)	1.137	0.256	400.989	12	< 0.001	97.007	−8.75049	0.14061
Zn	9	470/448	−0.283 (−0.758 to 0.192)	−1.167	0.243	105.608	9	< 0.001	91.478	−3.32396	0.46203
Fe	12	950/748	−0.050 (−0.652 to 0.552)	−0.163	0.871	393.975	13	< 0.001	96.700	−5.65837	0.26679
Lactoferrin	2	293/73	−0.039 (−0.321 to 0.244)	−0.268	0.788	2.322	2	0.313	13.854	−3.88793	0.30498
Transferrin	6	261/179	−0.009 (−0.302 to 0.285)	−0.057	0.955	12.513	5	0.028	60.040	−1.18916	0.77055
SOD	12	361/315	−0.036 (−0.74 to 0.668)	−0.100	0.921	189.185	11	< 0.001	94.186	1.60248	0.65811
Albumin	5	351/260	−0.213 (−0.879 to 0.454)	−0.626	0.532	59.325	4	< 0.001	93.258	15.27407	0.11314
Glutathione peroxidase	6	361/266	−0.233 (−0.770 to 0.303)	−0.853	0.394	40.986	5	< 0.001	87.801	4.45168	0.11213
Vitamin E	6	674/576	−0.155 (−0.470 to 0.159)	−0.967	0.334	42.968	6	< 0.001	86.036	11.14974	0.01384
Ceruloplasmin	6	338/321	−0.237 (−0.806 to 0.332)	−0.815	0.415	60.272	5	< 0.001	91.704	−5.47445	0.46487
Triglycerides	7	934/934	−0.089 (−0.311 to 0.132)	−0.791	0.429	21.418	6	0.002	71.986	2.69910	0.02914
Glutathione	7	397/481	−0.535 (−0.884 to −0.185)	−3.000	0.003	31.418	6	< 0.001	80.903	3.64575	0.13875
T-C	11	1,321/1,247	−0.241 (−0.455 to −0.026)	−2.199	0.028	56.529	10	< 0.001	82.310	2.42409	0.12460
LDL-C	11	1,281/1,259	−0.173 (−0.408 to 0.062)	−1.445	0.148	68.155	10	< 0.001	85.327	2.99418	0.07747
HDL-C	9	1,177/1,166	−0.041 (−0.183 to 0.101)	−0.562	0.574	17.323	8	0.027	53.818	1.82591	0.07727

*df, degrees of freedom; PD, Parkinson's Disease; 8-OHdG, 8-hydroxyguanosine; MDA, malondialdehyde; TBARS, thiobarbituric acid-reactive substances; SOD, superoxide dismutase; T-C, total-cholesterol; LDL-C, low-density lipoprotein-cholesterol; HDL-C, high-density lipoprotein-cholesterol*.

### Association of PD with CSF oxidative stress markers

Only five CSF oxidative stress markers, 8-OhdG, Mn, Cu, Zn, and Fe were included in the meta-analysis. Random-effects meta-analysis did not show significant differences between PD patients and HC subjects for these CSF oxidative stress markers (Table 2). However, there was a trend that CSF 8-OhdG levels were increased in patients with PD compared with HC subjects (*p* = 0.088, Table 2).

**Table 2:** Summary of Comparative Outcomes for Measurements of CSF Marker Levels.

**Cytokine**	**No. of studies**	**No. with PD/controls**	**Main effect**		**Heterogeneity**					**Publication bias**	
			**Hedges *g* (95% CI)**	***z* score**	***p* value**	**Q statistic**	***df***	***p* value**	***I*^2^ statistic**	**Egger intercept**	***p* value**
8-OHdG	3	86/71	0.816 (−0.120 to 1.751)	1.708	0.088	15.561	2	< 0.001	87.147	12.25750	0.31541
Mn	4	94/87	0.135 (−0.398 to 0.667)	0.496	0.620	8.938	3	0.03	66.437	−2.59509	0.66922
Zn	3	86/65	0.066 (−1.156 to 1.289)	0.106	0.915	24.043	2	< 0.001	91.681	10.74893	0.44572
Cu	4	117/100	0.325 (−0.332 to 0.982)	0.969	0.333	15.733	3	0.001	80.932	7.02799	0.22078
Fe	6	361/175	−0.131 (−0.606 to 0.344)	−0.540	0.589	35.582	6	< 0.001	83.138	−2.95205	0.54934

*df, degrees of freedom; PD, Parkinson's Disease; CSF, cerebrospinal fluid; 8-OHdG, 8-hydroxyguanosine*.

### Investigation of heterogeneity

In peripheral blood, significant heterogeneity was found for 21 of the 22 oxidative stress markers. Ferritin, uric acid, 8-OhdG, MDA, nitrite, Catalase, Mn, Cu, Zn, Fe, SOD, albumin, glutathione peroxidase, vitamin E, ceruloplasmin, triglycerides, glutathione, total-cholesterol, LDL-cholesterol showed high levels of heterogeneity, whereas transferrin and HDL-cholesterol showed moderate levels of heterogeneity. In addition, lactoferrin showed no significant heterogeneity. In CSF, all the five oxidative stress markers showed significant heterogeneity among studies.

Due to the limited number of studies analyzing CSF oxidative stress markers, we next attempted to adjust confounders that explained the heterogeneity among studies for blood oxidative stress markers. We considered both categorical variables (source of sampling, assay type and medication status) and continuous variables (age, sex, publication year, disease duration, and disease severity). Given the information on medication status, disease severity and disease duration were limited (eTable), and only a few number of studies analyzed the markers by ELISA method. We therefore performed subgroup analyses based on the source of sampling (serum and plasma), and meta-regression analyses based on publication year, age, and gender. In addition, for the oxidative stress markers significantly associated with PD, the number of studies were less than ten for four markers, we thus conducted subgroup and meta-regression analyses on oxidative stress markers MDA, uric acid, and total-cholesterol.

The heterogeneity was unchanged for plasma (*Q* = 34.296; *P* < 0.001; *I*^2^ = 91.253; 4 studies) and serum (*Q* = 71.204; *P* < 0.001; *I*^2^ = 92.978; 6 studies) studies, and the significant association between elevated TBARS levels and PD was retained in plasma studies, but not in serum studies (eFigure 4). For total-cholesterol, the heterogeneity was reduced 20% (*Q* = 13.499; *P* = 0.020; *I*^2^ = 62.822; 6 studies), and the significant association between reduced total-cholesterol levels and PD was retained for the serum studies. In addition, the heterogeneity was slightly reduced (*Q* = 16.498; *P* = 0.002; *I*^2^ = 75.755; 5 studies) and the significant association between reduced total-cholesterol levels and PD was lost for the plasma studies (eFigure 5). Considering only 2 out 18 studies analyzed plasma uric acid levels, we did not perform subgroup analysis on uric acid.

Meta-regression analyses for MDA showed that gender (regression coefficient [SE], 0.03037[0.00613]; 95% CI, 0.01835 to 0.04239; *P* < 0.001) and publication year (regression coefficient [SE], 0.05015 [0.00786]; 95% CI, 0.03475 to 0.06555; *P* < 0.001) were positively associated with ES, and age was negatively associated with ES (regression coefficient [SE], −22120.07023 [0.00935]; 95% CI, −0.08855 to −0.05190; *P* < 0.001), as shown in eFigure 6A. Moreover, age had moderating effect on the outcome of the meta-analysis (regression coefficient [SE], 0.02680 [0.00994]; 95% CI, 0.00732 to 0.04628; *P* = 0.00702) for studies measuring blood total-cholesterol levels, but not gender and publication year (eFigure 6B). For uric acid, the results showed that gender was significantly associated with ES (regression coefficient [SE], 0.00839 [0.00276], 95% CI, 0.00297 to 0.01381; *P* < 0.01), whereas age and publication year had no moderating effect on the outcome of the meta-analysis (eFigure 6C).

Sensitivity analysis demonstrated that no single study influenced the significant difference on blood MDA and uric acid levels between PD patients and HC subjects. However, we found that a single study could influence the statistically significant difference on blood total-cholesterol levels between PD patients and HC subjects.

No significant risk for publication bias was detected in the included studies as demonstrated by the Egger's (Tables 1, 2).

## Discussion

In this study, we have undertaken the first meta-analysis to investigate the alterations of circulating oxidative stress markers in patients with PD compared with HC subjects. We included 80 studies with 7,212 PD patients and 6,037 HC subjects measuring 22 blood oxidative stress markers, and reported the levels of oxidative stress (injury) makers 8-OhdG and MDA were elevated in patients with PD. As a reactive nitrogen species, nitrite can directly or indirectly oxidize or destroy DNA and proteins (Surendran and Rajasankar, 2010). Our data showed the higher level of nitrite in PD patients relative to the control, suggesting abnormal production of nitrite is closely related to Parkinson's diseases. Levels of ferritin, which is considered to reduce ROS accumulation upon oxidative stress, were also increased in PD patients compared with controls. Moreover, the levels of enzymatic antioxidant catalase and non-enzymatic antioxidants uric acid, glutathione and total-cholesterol were significantly decreased in patients with PD compared with controls. For those blood oxidative stress markers significantly associated with PD, the result associated with the ES was the largest for MDA, whereas the ESs ranged from small to large for other individual oxidative stress marker. In CSF, due to the limited number of studies with small sample size, this meta-analysis could only show a tread that 8-OhdG was associated with PD. Although the literature has yielded inconsistent results between studies and for individual oxidative stress markers, this study utilized meta-analytic technique provides strong clinical evidence of disruption between ROS generation and the body's antioxidant defense system in patients with PD, strengthening the clinical evidence that patients with PD have increased oxidative stress.

In addition to PD, increased oxidative stress has been also suggested to be involved in other neurological diseases such as Alzheimer's disease (AD), depression, and schizophrenia. A previous meta-analysis has been performed to address the inconsistent data in the literature regarding oxidative stress marker levels in patients with AD (Schrag et al., 2013). Similar to the findings of our present meta-analysis, levels of lipid peroxidation marker TBARS and non-enzymatic antioxidant uric acid were dysregulated in patients with AD. However, the enzymatic antioxidant catalase, with the present meta-analysis showed significant association with PD, was not associated with AD. Meta-analyses from depression (Liu et al., 2015) and schizophrenia (Flatow et al., 2013) studies showed similar dysregulation of certain oxidative stress markers in depression and schizophrenia patients as compared to AD and PD patients, whereas changes of some oxidative stress markers are unique in particular neurological disorders. Furthermore, none of the previous meta-analyses analyzed CSF oxidative stress markers levels in patients with neurological disorders. Here we have performed a meta-analysis of CSF oxidative stress markers in patients with PD compared with controls, and showed a non-significant association between 8-OhdG and PD. Although increased oxidative stress has been considered to be a major feature in various neurological diseases, these above results suggest that neurological diseases including AD, PD, depression, and schizophrenia have shared and distinct oxidative stress responses.

The levels of heterogeneity ranged from small to high for the individual oxidative stress markers in the meta-analysis, and most of the 22 blood markers showed high levels of between-study heterogeneity. For the blood oxidative stress markers significantly associated with PD, all markers showed high levels of heterogeneity. The strength of this meta-analysis is that we have used subgroup and meta-regression analyses to adjust the confounders that explain the between-study heterogeneity. Here we performed subgroup and meta-regression analyses on uric acid, total-cholesterol and TBARS because they had sufficient number of studies to explore the potential variables that contribute to the heterogeneity. Our results suggested that sampling source, age, gender, and publication year had moderating effects on the outcome of the meta-analysis, indicating that clinical and methodological variables may confound the oxidative stress profile in patients with PD, therefore raising awareness that these relevant confounding factors should be controlled for future clinical studies. In addition, sensitivity analysis showed the significant differences for blood uric acid and TBARS levels between PD patients and HC subjects were not influenced by any single study, suggesting the robustness of the outcome of meta-analysis. Although a single study could influence the statistically significant difference on blood total-cholesterol levels between PD patients and controls, this is not surprising given that the ES associated with total-cholesterol is relatively small.

Although this meta-analysis included 80 studies with large sample size providing strong clinical evidence that PD patients were accompanied by increased oxidative stress, there are several limitations in this study. The first limitation is that it is unclear whether the blood levels of oxidative stress markers in patients with PD associated with their levels in CSF. However, our meta-analysis suggested that the levels of DNA damage marker 8-OhdG were increased both in blood and CSF of PD patients, although it did not reach statistical significance for the association between CSF 8-OhdG and PD. This is likely due to the high levels of between-study heterogeneity and the smaller sample size for CSF 8-OhdG studies (3 studies with 86 patients and 71 controls) as compared with blood 8-OhdG studies (4 studies with 349 patients and 197 controls), making the observation of significant association difficult in CSF. It should be noted that CSF 8-OhdG would be significantly associated with PD if we choose fixed effect meta-analysis (*p* < 0.001) instead of a random effects model (a conservative model). Therefore, the CSF oxidative stress marker profile is likely to be similar with the blood oxidative stress marker profile, but future studies on CSF oxidative stress marker levels in PD patients are necessary to substantiate this idea.

Second, most of the studies included in this meta-analysis did not provide detailed information on disease severity, disease duration and medication status, which precluded us from analyzing whether these clinical variables affect the outcome of the meta-analysis. Several studies included in this meta-analysis suggested these variables affected the oxidative stress marker levels. Data from Gokce Cokal et al. showed that blood TBARS level was inversely correlated with the duration of PD (Gökçe Çokal et al., 2017), and Chen et al. demonstrated that blood TBARS level was negatively correlated with disease severity and age in PD patients (Chen et al., 2009), supporting our meta-regression analysis result that age is a confounding factor for the outcome of the meta-analysis analyzing blood TBARS levels. However, since older ages were likely to be associated with longer disease duration or medication use in PD patients, these suggest another possibility that the moderating effect of age on MDA level was secondary to disease severity, disease duration or duration of medication use. In addition, one study demonstrated strong and significant inverse correlations of uric acid with PD disease duration and daily levodopa dosage (Andreadou et al., 2009), whereas another study only found a negative correlation of uric acid with daily levodopa dosage in men (Jesús et al., 2013). Nevertheless, these results highlight the need for continued work on oxidative stress marker levels in PD patients, with control for disease severity, disease duration and medication status, and may provide markers to monitor disease progression in PD patients.

The third limitation of this meta-analysis is that we only included English-language articles, and this may generate publication bias. However, given the very limited number of non-English articles in this field, it is unlikely that this would significantly affect the outcome of our meta-analysis.

## Conclusions

Our meta-analysis findings demonstrated elevated peripheral blood concentrations of 8-OhdG, MDA, nitrite and ferritin, and reduced blood levels of catalase, uric acid, glutathione, total-cholesterol in patients with PD. This finding strengthens the clinical evidence that PD is accompanied by increased oxidative stress response, and manipulation of oxidative stress marker concentrations should be investigated for potential therapeutic strategies of the disease.

## Author contributions

YC and QL conceived and designed the study, ZW, XiaL and XixL collected the data. All the authors analyzed and interpreted the data. YC wrote the manuscript with editing from QL.

### Conflict of interest statement

The authors declare that the research was conducted in the absence of any commercial or financial relationships that could be construed as a potential conflict of interest.
