# Supplementary material for: Oxidative Stress in Parkinson's Disease: A Systematic Review and Meta-Analysis
Source: Front Mol Neurosci. 2018 Jul 5;11:236. doi: 10.3389/fnmol.2018.00236 (PMC6041404; doi:10.3389/fnmol.2018.00236)
Supplement: Supplementary file 1 [file DataSheet1.docx]

# Supplementary Online Content

**eTable:** Characteristics of included studies measuring peripheral blood biomaker concentrations

**eFigure 1:** Forest plot for random-effects meta-analysis for catalase, ferritin and uric acid

**eFigure 2:** Forest plot for random-effects meta-analysis for hydroxyguanosine and total-cholesterol

**eFigure 3:** Forest plot for random-effects meta-analysis for glutathione , nitrite and TBARS

**eFigure 4:** Sub-group analysis stratified by source of sampling for TBARS

**eFigure 5:** Sub-group analysis stratified by source of sampling for total-cholesterol

**eFigure 6:** Meta-regression

# eReference:^[1-80](#_ENREF_1)^

**eTable:Characteristics of included studies measuring peripheral blood biomaker concentrations**

| **Study/Year** | **Cytokines Measured** | **Country** | **Samples (PD/HC)** | **Gender**  **(% Male) (PD/HC)** | **Mean**  **Age (PD/HC)** | **Mean PD duration** | **Mean**  **H & Y scale** | **Mean UPDRS** | **Sample Source** | **Diagnosis** | **Assay type** | **Medication** |
| --- | --- | --- | --- | --- | --- | --- | --- | --- | --- | --- | --- | --- |
| Abe et al. 2003 | 8-OHG | Japan | 24/15 | 50/46.66 | 63.3/62.3 | 2.80 | 2.5 | NA | Serum  &CSF | Koller’s criteria/H&Y stage | HPLC | NA |
| Ahlskog et al. 1995 | MDA | USA | 43/15 | 72.09/20 | 61.9/61.4 | 6.30 | NA | NA | Serum | Diagnosti and Statistical Manual of Mental Disorders | TBA method | Y(part) |
| Ahmed et al.  2010 | Cu, Fe, Mn,Zn | India | 45/42 | 57.77  /59.52 | 57.62  /55.62 | NA | NA | NA | Serum | UPDRS/H&Y stage | ICP-AES , ICP-MS | NA |
| Alimonti et al.  2007 | Cu.Fe,Zn,Mn | Italy | 71/124 | 74.64/65.  32 | 65.5/44.8 | 4.60 | 1.55 | NA | Serum | H&Y stage, London Brain  Bank Criteria | Coupled plasma atomic  emission spectrometry. | Y(part) |
| Andican et al.  2012 | HDL-C,  LDL-C,TG, TC | Turkey | 45/25 | 55.55/60 | 63.6/60.2 | 6.40 | NA | NA | plasma | H&Y stage | Enzymatic methods | Y |
| Andreadou et  al. 2009 | UA | Greece | 43/47 | 51.16/  53.19 | 67.23/  67.23 | 3.00 | 2.5 | 25 | Serum | UKPD criteria/UPDRS/H&Y  stage | Olympus AU 600 random access  analyzer | Y(part) |
| Annanmaki et al. 2007 | Ferritin, TRF,Fe,Cp,UA | Finland | 40/29 | 58/45 | 60.8/60.2 | NA | NA | NA | plasma/ serum | UKPD criteria | Ferritin: immunochemiluminometric  assay  TRF: immunochemical assay Fe:spectrophotometric method Cp:NA  UA: enzymatic assay | NA |
| Annanmaki et al. 2011 | UA | Finland | 28/12 | 61/33 | 60.3/63.4 | 3.70 | 1.6 | NA | plasma | UKPD criteria,UPDRS,H&Y stage | NA | Y(part) |

| **Study/Year** | **Cytokines Measured** | **Country** | **Samples (PD/HC)** | **Gender (% Male)**  **(PD/HC)** | **Mean Age**  **(PD/HC)** | **Mean PD duration** | **Mean H & Y**  **scale** | **Mean UPDRS** | **Sample Source** | **Diagnosis** | **Assay type** | **Medication** |
| --- | --- | --- | --- | --- | --- | --- | --- | --- | --- | --- | --- | --- |
| Arnal et al.  2010 | Cu, Cp | Argentina | 87/134 | 42.52/50 | 70.0/63.1 | 6.50 | NA | NA | Plasma | MMSE,H&Y stage | Spectrophotometric method | NA |
| Baillet et al. 2010 | MDA,SOD,GS  H-px, CAT，Total GSH,GSH,GS SG | France | 24/30 | 70.83/36.  66 | 57.8/39.4 | NA | NA | NA | Plasma | UPDRS/H&Y stage | MDA:HPLC  SOD,GSH-px,CAT: Enzymatic method  Total GSH: the Ellman’s test GSH,GSSG:  spectrophotometric method | NA |
| Bharucha et al. 2008 | Cp,Cu,Ferritin | USA | 91/40 | 75.82/65 | 53.9/49.0 | 9.20 | NA | NA | Serum | UKPD criteria | Cp:nephelometric immunoturbidimetric assay  Cu,Ferritin:NA | Y |
| Bogdanov et al. 2008 | 8-OHdG,  reduced GSH | USA | 66/25 | 59/33 | 66.0/61.5 | 7.10 | NA | NA | Plasma | NA | LCECA | Y (Part) |
| Boll et al.  1999 | Cu | Mexico | 49/26 | 65/46 | 61.3/58.7 | 5.25 | 2.16 | 29.07 | CSF | H&Y stage/UPDRS | AAS | Y(part) |
| Bostantjopoul ou et al. 2005 | NO,SOD | Greece | 57/40 | 61.4/NA | 58.0/NA | 8.10 | 2.7 | 30.3 | plasma | UPDRS/H&Y stage | NO:colorimetric assay  SOD:the Ransod reagents method | Y(part) |
| Brewer et al.  2010 | Zn | USA | 30/29 | 60/31 | 67.4/68.6 | NA | NA | 17.8 | Serum | PDBBC/UPDRS | AAS | NA |
| Cabrera  -Valdivia et al. 1994 | Fe, TRF,ferritin | Spain | 68/68 | 52.94/47.  05 | 65.8/65.8 | 5.30 | 2.5 | NA | Serum | H & Y stage//UPDRS | Fe,ferritin:BM/Hitachi system 704  TRF:kinetic  immunonephelometry | Y(part) |

| **Study/Year** | **Cytokines Measured** | **Country** | **Samples (PD/HC)** | **Gender (% Male)**  **(PD/HC)** | **Mean Age**  **(PD/HC)** | **Mean PD duration** | **Mean H & Y**  **scale** | **Mean UPDRS** | **Sample Source** | **Diagnosis** | **Assay type** | **Medication** |
| --- | --- | --- | --- | --- | --- | --- | --- | --- | --- | --- | --- | --- |
| Chen et al. 2009 | 8-OHdG,GSH-  Px,MDA,vitami n E | Taiwan | 211/135 | 56.4/48.2 | 67.3/68.4 | NA | NA | NA | Plasma | H & Y stage | 8-OHdG:HPLC-ECD analysis GSH-px:NA  MDA,vitamin E :HPLC | Y (Part) |
| Constantinesc  u et al. 2013 | UA | Sweden | 6/18 | 61.1/66.6 | 51/68 | 4.00 | 2 | NA | Serum  &CSF | UKPD criteria clinical | enzymatic method | Y |
| Cristalli et al. 2012 | NOx,Cu,GSS G,GSH | Argentina | 87/134 | 42.52/50 | 70.0/63.1 | 6.5 | NA | NA | plasma | MMSE/Global Deterioration Scale/H & Y stage | GSSG,GSH: enzymatic recycling method  NOx:NA  Cu:Spectra AA 300/400 analysis | NA |
| Cubukcu et al.  2016 | NO | Turkey | 32/32 | 50/40.62  5 | 66.78/64.  64 | 58.90 | 2.06 | 25.75 | Serum | H & Y stage/UPDRS | Griess method | Y(part) |
| de Farias et al. 2016 | MDA,NOx,CA T,SOD | Brazil | 56/56 | 48.2/44.4 | 70.3/69.7 | 6.50 | NA | NA | Plasma | UKPD/H & Y stage | MDA:HPLC  NOx:semiautomated method CAT:enzymic method SOD:enzymic method | Y |
| Du et al. 2012 | TC,LDL-C | USA | 40/29 | 57.5/41.4 | 60.7/59.6 | 4.20 | 1.5 | 23.4 | serum | UPDRS | Biochemical methods | Y |
| Duran et al. 2011 | HDL-C,LDL-C, TC,TG | Spain | 95/60 | 41.04/50 | 64.75/48.  02 | 9.18 | 1.95 | NA | Plasma | UKPD criteria/H & Y stage | HDL-C,TC,TG:automatic analyzer  LDL-C:Friedewald formula | Y(part) |
| Forte et al. 2004 | Cu,Zn,Fe,Mn | Italy | 26/13 | 92.3/46.1  5 | 64.9/63.8 | 4.80 | NA | NA | Serum  &CSF | London Brain Bank Criteria/UPDRS/H & Y  stage | Cu,Zn,Fe:ICP-AES Mn:SF-ICP-MS | Y |
| Forte et al.  2005 | Cu,Fe,Mn,Zn | Italy | 71/44 | 74.64/75 | 65.5/51.9 | 4.62 | NA | NA | Serum | London Brain Bank  Criteria/H & Y stage | NA | Y(part） |

| **Study/Year** | **Cytokines Measured** | **Country** | **Samples (PD/HC)** | **Gender (% Male)**  **(PD/HC)** | **Mean Age**  **(PD/HC)** | **Mean PD duration** | **Mean H & Y**  **scale** | **Mean UPDRS** | **Sample Source** | **Diagnosis** | **Assay type** | **Medication** |
| --- | --- | --- | --- | --- | --- | --- | --- | --- | --- | --- | --- | --- |
| Fukushima et al. 2011 | Fe,Cu,Mn,Zn,v itamin E | Japan | 71/71 | 57.74/57.  74 | 63.7/63.4 | NA | NA | NA | Serum | UKPD criteria | Fe,Cu,Zn:coupled plasma atomic emission spectrometry  Mn:atomic absorption spectrometry.  vitamin E:fluorometric method | NA |
| Gangania et al. 2017 | Fe,Cu,Cp | India | 35/33 | NA | 57/50 | NA | NA | NA | Serum | UPDRS/MMSE | Fe:ferene method  Cu:colorimetric kit Cp:turbidimetric assay | NA |
| Gatto et al.  1996 | NO,SOD,CAT | Argentina | 12/10 | 66.66/NA | 61.1/58 | 11 | NA | NA | plasma | UKPD criteria/H & Y stage | Spectrofluorimeter method | Y(part) |
| Gazzaniga et  al. 1992 | Cu, Fe,Mn | Italy | 11/22 | 90.9/90.9 | 64.9/NA | 3.39 | NA | NA | CSF | H & Y stage/Columbia  University rating scale | AAS | Y(part) |
| Gironi et al. 2011 | MDA,GSH | Italy | 20/66 | 65/31.81 | 72.1/70.4 | NA | NA | NA | Serum | the criteria by Emre et al | MDA:isocratic HPLC system  GSH:HPLC | Y(part) |
| Gokce Cokal et al. 2017 | MDA,GSH-Px, SOD | Turkey | 29/32 | 55.2/40.6 | 66.55/64.  64 | 5.46 | 2.28 | 27.5 | Serum | H & Y stage/UPDRS/UK Parkinson’s disease Brain Bank criteria | MDA: TBARS method  GSH-Px:NADPH absorbance method  SOD:NBT reduction rate method | Y |
| Gonzalez-Ara mburu et al.  2013 | UA | Spain | 365/132 | 57.3/31.8 | 70.2/65.2 | 5.4 | NA | NA | Serum | UKPD criteria | ADVIA 2400 chemistry system | NA |

| **Study/Year** | **Cytokines Measured** | **Country** | **Samples (PD/HC)** | **Gender (% Male)**  **(PD/HC)** | **Mean Age**  **(PD/HC)** | **Mean PD duration** | **Mean H & Y**  **scale** | **Mean UPDRS** | **Sample Source** | **Diagnosis** | **Assay type** | **Medication** |
| --- | --- | --- | --- | --- | --- | --- | --- | --- | --- | --- | --- | --- |
| Grau et al. 2001 | LF,Ferritin,TR F | Germany | 23/15 | 65.21/66.  66 | 59/55 | 7.40 | 2.5 | NA | Plasma | H & Y stage | LF:enzyme immunoassay Ferritin: electrochemoluminescence  TRF: nephelometry | Y(part) |
| Gregório et al. 2013 | Total-C,  LDL-C,HDL-C, TG | Brazil | 86/169 | 62/48 | 69.2/71.7 | NA | NA | NA | plasma | Neuroimaging method | NA | NA |
| Gruden et al.  2012 | MDA,SOD,  CAT, GSH | Russia | 32/26 | 62.5/73.0  7 | 60.8/63.0 | NA | 2.1 | 23.3 | Serum | H & Y stage/UPDRS | MDA: TBA test  SOD, CAT, GSH:NA | Y |
| Guo et al.  2015 | TC,LDL-C,  HDL-C, TG | China | 555/555 | 57.11/57.  11 | 62.2/62.4 | 3.80 | 2.5 | 27 | Serum | UKPD criteria /H & Y  stage/UPDRS | NA | NA |
| Håglin et al. 2016 | TRF, Albumin | Sweden | 75/24 | 54.66/50 | 68.9/68.2 | NA | 2.03 | 34.6 | plasma | H & Y stage/UPDRS/UKPD criteria | TRF:Vitros TRFRN reagent Albumin: Vitros ALB Slides | Y(part) |
| Hemmati-Dina rvand et al.  2017 | Ferritin,UA | Iran | 40/40 | 67.5/55 | 65.70/64.  35 | 3.78 | NA | NA | Serum | UKPD criteria/H & Y stage | Ferritin:ELISA,Luminex-100 instrument  UA:Enzymatic-Colorimetric  Uricase assay | NA |
| Hozumi et al.  2011 | Cu Fe Mn Zn | Japan | 20/15 | 45/40 | 68.7/48.4 | NA | NA | NA | CSF | British Brain Bank criteria | ICP-MS | NA |
| Huang et al.  2007 | LDL-C,HDL-C,  TC | USA | 124/112 | 55.6/44.6 | 67.9/65.7 | 4.2 | NA | NA | Serum | UPDRS | NA | Y(part) |
| Ikeda et al. 2011 | Urate,Ferritin, TG,TC,LDL-C | Japan | 119/120 | 47.05/50 | 73.4/72.9 | 6.90 | 3.2 | NA | Serum | UKPD criteria | Urate,Ferritin, TG,TC:Olympus AU600 chemistry analyzer  LDL-C: the Friedewald formula | Y |

| **Study/Year** | **Cytokines Measured** | **Country** | **Samples (PD/HC)** | **Gender (% Male)**  **(PD/HC)** | **Mean Age**  **(PD/HC)** | **Mean PD duration** | **Mean H & Y**  **scale** | **Mean UPDRS** | **Sample Source** | **Diagnosis** | **Assay type** | **Medication** |
| --- | --- | --- | --- | --- | --- | --- | --- | --- | --- | --- | --- | --- |
| Jesus et al.  2013 | UA | Spain | 161/178 | 55.9/60.6  7 | NA | NA | NA | NA | Serum | H & Y stage | NA | Y |
| Jiménez-Jimé nez et al.  1998 | Fe, Cu, Mn,Zn, Albumin | Spain | 37/37 | 37.83/43.  24 | 65.7/62.4 | 7.00 | 2.9 | 39.3 | Serum  &CSF | H & Y stage/UPDRS | Fe, Cu, Mn,Zn:AAS  Albumin:NA | Y (Part) |
| Jimenez-Jime nez et al. 1992 | Zn, Cu,Cp, Albumin | Spain | 39/39 | 51.28/48.  71 | 67.3/66.2 | 6.00 | 2.9 | NA | Serum | Unified Parkinson’s Disease Rating Scale/H & Y stage | Zn, Cu:flame AAS  Cp,Albumin: immunonephelometry | Y (Part) |
| Kalra et al.  1992 | SOD,GSH-Px | Canada | 18/19 | 50/52.63 | 59.3/64.2 | NA | NA | NA | Serum | NA | Enzymatic assay | NA |
| Kalra et al.  1992 | MDA | Canada | 22/28 | 59.09/50.  00 | 59.4/63.9 | NA | NA | NA | Serum | NA | TBA method | NA |
| Kikuchi et al.  2002 | 8-OHdG | Japan | 48/22 | 50/40.9 | 65.7/60.5 | 8.5 | 3.2 | NA | Serum  &CSF | H & Y stage/Koller’s criteria | ELISA | Y(part) |
| Kim et al. 2014 | UA | America | 30/25 | 63.33/48 | 57.6/56.2 | 1.65 | 9 | 24.5 | Serum | UK Brain Bank criteria,UPDRS,H & Y stage | Enzymatic colorimetric test | NA |
| Kim et al.  2017 | TC, Albumin | Korea | 104/52 | 60.577/7  1.153 | 66.9/65.4 | 3.70 | 2.5 | 19.9 | Serum | H & Y stage/UPDRS | NA | Y |
| Kirbas et al. 2014 | LDL-C,HDL-C, TG,TC | Turkey | 42/40 | 64/60 | 59.3/57.0 | NA | NA | NA | Serum | PDBBC /H & Y stage | LDL-C:the Friedewald formula HDL-C,TG,TC:NA | NA |
| Kouti et al. 2013 | NO | Iran | 58/15 | 56.1/40 | 64.4/58.3 | 5.40 | NA | 28.83 | Serum | UPDRS | NO:Griess method | Y |

| **Study/Year** | **Cytokines Measured** | **Country** | **Samples (PD/HC)** | **Gender (% Male)**  **(PD/HC)** | **Mean Age**  **(PD/HC)** | **Mean PD duration** | **Mean H & Y**  **scale** | **Mean UPDRS** | **Sample Source** | **Diagnosis** | **Assay type** | **Medication** |
| --- | --- | --- | --- | --- | --- | --- | --- | --- | --- | --- | --- | --- |
| Kumudini et al. 2014 | MDA,GSH,Cu,  Fe,Mn | India | 150/170 | 71.33/68.  57 | 55.7/53.7  3 | 7.90 | NA | NA | plasma | UPDRS | MDA: TBA method  GSH: Ellman’s method Cu,Fe,Mn：ICP-MS | NA |
| Loeffler et  al.2017 | 8-OHdG | USA | 31/27 | 48/37 | 60.5/61.5 | 3.00 | NA | 27 | CSF | Gelb criteria/exome  sequencing/UPDRS | Antioxidant Assay Kit | NA |
| Lolekha et al.  2015 | UA | Thailand | 100/100 | 50/50 | 68.14/66.  31 | 4.44 | 2.33 | 23.41 | Serum | UKPD criteria/H & Y  stage/UPDRS | NA | Y (Part) |
| Medeiros et al.  2016 | Fe,Ferritin,TR  F | Brazil | 40/46 | 45/41 | 65.95/62.  30 | 8.57 | 2.5 | 44.26 | Serum | UKPD criteria/H & Y  stage/UPDRS | NA | NA |
| Miao et al.  2017 | UA | China | 220/110 | 55/54.55 | 68.098/6  9.480 | 6.079 | 2.305 | 23.147 | Serum | UKPD criteria | Automatic biochemical analyzer | NA |
| Molina et al.  1992 | MDA | Spain | 37/37 | 51.35/48.  64 | 67.2/66.2 | 5.90 | 2.8 | 33.2 | Serum | H & Y stage/UPDRS | Fluorometric method | Y(part) |
| Naduthota et al. 2017 | MDA | India | 72/72 | 80.55/80.  55 | 51.4/50.8 | 5.10 | 1.7 | 21.3 | Serum | UKPD criteria/UPDRS/H&Y stage | Spectrophotometric method | NA |
| Nicoletti et al.  2001 | Vitamin E | Italy | 54/93 | 59.25/53.  76 | 66.6/69.5 | NA | NA | NA | Plasma | UKPD criteria/UPDRS | HPLC | Y |
| Paraskevas et  al. 2003 | Vitamins E | Greece | 44/39 | 59.09/56.  41 | 60/59 | 5.30 | 3 | NA | plasma | H & Y stage | HPLC | NA |
| Qin et al.  2015 | UA | China | 425/459 | 33.64/35.  29 | 67.32/67.  84 | 3.50 | NA | NA | Serum | H & Y stage/ UKPD criteria | Clinical Analyzer 7600-ISE | N |
| Sakuta et al. 2017 | UA | Japan | 100/100 | 46/60 | 68.5/66.7 | 5.30 | 3.1 | 28.5 | Serum | UKPD criteria/H & Y stage/UPDRS criteria/UPDRS/H&Y stage | NA | NA |
| Sampat et al. | UA | USA | 20/20 | 50/50 | 66.5/64.7 | 7.10 | NA | 30.2 | Urine | H & Y stage/UPDRS | florescence-based kit | Y |

| **Study/Year** | **Cytokines Measured** | **Country** | **Samples (PD/HC)** | **Gender (% Male)**  **(PD/HC)** | **Mean Age**  **(PD/HC)** | **Mean PD duration** | **Mean H & Y**  **scale** | **Mean UPDRS** | **Sample Source** | **Diagnosis** | **Assay type** | **Medication** |
| --- | --- | --- | --- | --- | --- | --- | --- | --- | --- | --- | --- | --- |
| 2016 |  |  |  |  |  |  |  |  |  |  |  |  |
| Serra et al.  2001 | SOD,CAT | Argentina | 15/14 | 46.66/50 | 71.33/71.  21 | NA | NA | NA | Plasma | H & Y stage | Spectrophotometric method | Y |
| Shukla et al.  2006 | Nitrite,MDA | India | 21/20 | 76.19/NA | 57.7/NA | 2.7 | NA | 16.9 | CSF | UPDRS,H & Y stage | MDA:TBA method  NO:spectrophotometric | Y (Part) |
| Sterling et al.  2016 | LDL-C | USA | 64/64 | 59.38/50 | 62.7/61.3 | 4.4 | 1.7 | NA | plasma | MMSE,H & Y stage | Friedwald equation | Y |
| Sun et al.  2012 | UA | China | 411/396 | 50.36/51.  01 | 63.11/62.  13 | 5.73 | 2.55 | NA | Serum | H & Y stage/ UKPD  criteria | enzymatic assay | NA |
| Urakami et al.  1992 | SOD | Japan | 19/23 | 52.63/21.  73 | 66.6/66.4 | 5.20 | NA | NA | plasma | H & Y stage | Nitrite method | Y (Part) |
| van Kamp et  al. 1995 | TRF | Netherla  nds | 90/21 | 52.22/52.  38 | 67.2/61.5 | NA | NA | NA | Serum  &CSF | NA | Beckman Array automatic  nephelometer | Y(part) |
| Vieru et al.  2016 | UA | Turkey | 80/80 | 44/51 | 67.99/66.  51 | NA | NA | NA | Serum | H & Y stage/ UKPD  criteria/UPDRS | NA | Y |
| Vinish et al. 2011 | SOD,GSH-px , NO,MDA | India | 15/10 | NA | 46.5/43.4 | NA | NA | NA | whole blood/pl asma | UKPD criteria | SOD,GSH-px:enzymatic methods  NO:Griess method MDA:TBA method | NA |
| Wang et al. 2016 | Fe,NO | China | 145/30 | 50.60/50 | 57.81/61.  86 | 3.19 | NA | 24.09 | CSF | UKPD criteria/H & Y stage/UPDRS | Fe:ELISA  NO:chemical colorimetric method | Y |
| Wang et al.  2017 | UA, Albumin | China | 96/108 | 54.2/60.2 | 67.54/66.  34 | 3.78 | 3 | NA | Serum | H & Y stage/UKPD criteria | Automated biochemistry  analyzer | NA |
| Watfa et al. | SOD,Total | France | 20/15 | 55.0/26.7 | 77.5/78.7 | 4.40 | NA | NA | plasma | UKPD criteria | SOD,total GSH:enzymatic | Y |

| **Study/Year** | **Cytokines Measured** | **Country** | **Samples (PD/HC)** | **Gender (% Male)**  **(PD/HC)** | **Mean Age**  **(PD/HC)** | **Mean PD duration** | **Mean H & Y**  **scale** | **Mean UPDRS** | **Sample Source** | **Diagnosis** | **Assay type** | **Medication** |
| --- | --- | --- | --- | --- | --- | --- | --- | --- | --- | --- | --- | --- |
| 2011 | GSH,TG,TC,H DL-C,LDL-C |  |  |  |  |  |  |  |  |  | method  TG,TC,HDL-C: Olympus AU2700 Clinical Chemistry System  LDL-C: Friedwald formula |  |
| Younes-Mhen  ni et al. 2013 | Cu，Zn | Tunisia | 48/36 | 54.16/38.  88 | 65.8/59.7 | 6.30 | NA | NA | Serum | H & Y stage | AAS | Y (Part) |
| Yuan et al.  2016 | GSH,SOD,CA  T,GSH-Px, | China | 64/40 | 42.5/39.0  6 | 63.5/62.6 | 3.5 | 2.0 | NA | plasma | UPDRS/ UKPD brain bank  criteria/UPDRS | ELISA | N |
| Zhang et al.  2012 | UA | China | 534/614 | 63.85/62.  54 | 63.87/63.  56 | 4.13 | NA | NA | Serum | UKPD criteria | Automatic biochemistry analyzer | NA |
| Zhang et al. 2017 | TC,  LDL-C,HDL-C, TG | USA | 91/70 | 53.85/54.  29 | 65.8/63.5 | NA | NA | NA | plasma | MMSE | TC,HDL-C,TG:enzymatic methods  LDL-C: the Friedwald equation | NA |
| Zhao et al.  2014 | Cp | China | 46/46 | 55/54 | 60.17/61.  40 | NA | 1.8 | NA | Serum | UKPD criteria/H & Y  stage/UPDRS | immunonephelometry kits | NA |
| Zuo et al. 2016 | Fe,TRF,LF,Fer  ritin | China | 518/29 | 50.70/53.  11 | 60.85/61.  73 | 2.59 | 2.03 | 23.59 | Serum  &CSF | UKPD criteria/UPDRS/H&Y stage | ELISA | NA |

**Abbreviations**: PD, Parkinson's Disease; HC, Healthy Controls; Fe, iron; Cu, copper; Cp, Ceruloplasmin; UA, Uric acid; TRF, Transferrin; LF, Lactoferrin; TC, Total Cholesterol; NO, nitric oxide; CAT, CATALASE; TG, Triglycerides; LDL-C, low density lipoprotein cholesterol; HDL-C; high density lipoprotein cholesterol; SOD, Superoxide dismutase; GSH, Glutathione; Mn, manganese; TBARS, thiobarbituric acid-reactive substances; NADPH,nicotinamide adenine dinucleotide phosphate; NBT, nitro blue tetrazolium; UKPD, United Kingdom PD Society Brain Bank; TBA, thiobarbituric acid; AAS, atomic absorption spectrophotometer; ICP-AES, Inductively Coupled Plasma Atomic Emission Spectrometry; SF-ICP-MS, Sector Field Inductively Coupled Plasma Mass Spectrometry; MMSE, Mini-Mental State Examination; HPLC-ECD, high performance liquid chromatography-electrochemical detector; LCECA, high performance liquid chromatography coupled with electrochemical coulometric array detection; H&Y, Hoehn and Yahr scale; UPDRS, Unified Parkinson's Disease Rating Scale; MMSE, Mini-Mental State Exam; PDBBC, Parkinson's Disease Brain Bank Criteria; Y, Yes; N, No; NA, not available.

**Catalase**

**eFigure 1**

1. **Study name Statistics for each study Hedges's g and 95% CI Hedges's Lower Upper**

|  | **g** | **limit** | **limit** | **p-Value** |  | | | |
| --- | --- | --- | --- | --- | --- | --- | --- | --- |
| Baillet et al. 2010 | -0.030 | -0.559 | 0.499 | 0.912 |  |  |  |  |
| Serra et al. 2001 | 0.325 | -0.388 | 1.038 | 0.371 |  |  |  |  |
| Gatto et al. 1996 | -1.695 | -2.645 | -0.745 | 0.000 |  |  |  |  |
| Gruden et al. 2012 | -0.211 | -0.723 | 0.301 | 0.420 |  |  |  |  |
| Yuan et al. 2016 | -1.220 | -1.645 | -0.794 | 0.000 |  |  |  |  |
| de Farias et al. 2016 | -0.628 | -1.005 | -0.251 | 0.001 |  |  |  |  |
|  | -0.548 | -1.051 | -0.044 | 0.033 |  |  |  |  |
|  |  |  |  |  | **-2.00** | **-1.00** | **0.00 1.00** | **2.00** |

# Ferritin

**catalase decreased in PD catalase increased in PD**

### Study name Statistics for each study Hedges's g and 95% CI

|  | **Hedges's g** | **Lower limit** | **Upper limit** | **p-Value** |  |  |  |  |  |  | |
| --- | --- | --- | --- | --- | --- | --- | --- | --- | --- | --- | --- |
| Grau et al. 2001 | 0.080 | -0.557 | 0.717 | 0.806 |  |  |  |  |  |  |  |
| Cabrera-Valdivia et al. 1994 | 0.056 | -0.278 | 0.390 | 0.743 |  |  |  |  |  |  |  |
| Ikeda et al. 2011 | 0.399 | 0.143 | 0.654 | 0.002 |  |  |  |  |  |  |  |
| Bharucha et al. 2008 | 0.384 | -0.151 | 0.919 | 0.159 |  |  |  |  |  |  |  |
| Hemmati-Dinarvand et al. 2017 | 7.217 | 6.017 | 8.416 | 0.000 |  |  |  |  |  |  |  |
| Annanmaki et al. 2007 | -0.090 | -0.563 | 0.383 | 0.710 |  |  |  |  |  |  |  |
| Medeiros et al. 2016 | -0.145 | -0.566 | 0.275 | 0.498 |  |  |  |  |  |  |  |
| Zuo1 et al. 2016 | -0.079 | -0.481 | 0.323 | 0.701 |  |  |  |  |  |  |  |
| Zuo2 et al. 2016 | -0.047 | -0.444 | 0.350 | 0.816 |  |  |  |  |  |  |  |
|  | 0.659 | 0.055 | 1.264 | 0.033 |  |  |  |  |  |  |  |
|  |  |  |  |  | **-2.00** | **-1.00** |  | **0.00** |  | **1.00** | **2.00** |

**ferritin decreased in PD ferritin increased in PD**

# eFgure 1

**C**

**Uric acid**

**Study name Statistics for each study Hedges's g and 95% CI**

|  | **Hedges's g** | **Lower limit** | **Upper limit** | **p-Value** |  | | | | | |
| --- | --- | --- | --- | --- | --- | --- | --- | --- | --- | --- |
| Zhang et al. 2012 | -0.807 | -0.928 | -0.687 | 0.000 |  |  |  |  |  |  |
| Ikeda et al. 2011 | -0.651 | -0.911 | -0.392 | 0.000 |  |  |  |  |  |  |
| Andreadou et al. 2009 | -0.271 | -0.683 | 0.141 | 0.197 |  |  |  |  |  |  |
| Jesus et al. 2013 | -0.423 | -0.638 | -0.208 | 0.000 |  |  |  |  |  |  |
| Sun et al. 2012 | -0.466 | -0.605 | -0.326 | 0.000 |  |  |  |  |  |  |
| Constantinescu et al. 2013 | 0.000 | -0.892 | 0.892 | 1.000 |  |  |  |  |  |  |
| Hemmati-Dinarvand et al. 2017 | -0.128 | -0.563 | 0.306 | 0.562 |  |  |  |  |  |  |
| Qin et al. 2015 | -0.983 | -1.123 | -0.844 | 0.000 |  |  |  |  |  |  |
| Lolekha et al. 2015 | -0.383 | -0.662 | -0.105 | 0.007 |  |  |  |  |  |  |
| Sampat et al. 2016 | -0.164 | -0.772 | 0.445 | 0.598 |  |  |  |  |  |  |
| Vieru et al. 2016 | -0.883 | -1.206 | -0.559 | 0.000 |  |  |  |  |  |  |
| Wang et al. 2017 | -0.462 | -0.740 | -0.185 | 0.001 |  |  |  |  |  |  |
| Sakuta et al. 2017 | -0.664 | -0.948 | -0.380 | 0.000 |  |  |  |  |  |  |
| Miao et al. 2017 | -0.450 | -0.681 | -0.219 | 0.000 |  |  |  |  |  |  |
| Kim et al. 2014 | -0.682 | -1.220 | -0.143 | 0.013 |  |  |  |  |  |  |
| Annanmaki et al. 2007 | -0.530 | -1.011 | -0.049 | 0.031 |  |  |  |  |  |  |
| Annanmaki et al. 2011 | -0.311 | -0.978 | 0.355 | 0.360 |  |  |  |  |  |  |
| Gonzalez-Aramburu et al. 2013 | -0.033 | -0.231 | 0.166 | 0.747 |  |  |  |  |  |  |
|  | -0.500 | -0.649 | -0.350 | 0.000 |  |  |  |  |  |  |
|  |  |  |  |  | **-2.00** | **-1.00** |  | **0.00** | **1.00** | **2.00** |

**uric acid decreased in PD uric acid increased in PD**

Forest plot for random-effects meta-analysis on differences in blood catalase, ferritin and uric acid concentrations between Parkinson's Disease (PD) patients and healthy cognitive control (HC) . The sizes of the squares are proportional to study weight. CI, confidence interval.

**Hydroxyguanosine**

# eFigure 2

**A**

**Study name Statistics for each study Hedges's g and 95% CI**

|  | **Hedges's g** | **Lower limit** | **Upper limit** | **p-Value** |  | | | | |
| --- | --- | --- | --- | --- | --- | --- | --- | --- | --- |
| Bogdanov et al. 2008 | 0.293 | -0.166 | 0.751 | 0.210 |  |  |  |  |  |
| Chen et al. 2009 | 1.416 | 1.176 | 1.656 | 0.000 |  |  |  |  |  |
| Kikuchi et al. 2002 | 1.147 | 0.613 | 1.681 | 0.000 |  |  |  |  |  |
| Abe et al. 2003 | 0.123 | -0.510 | 0.755 | 0.704 |  |  |  |  |  |
|  | 0.772 | 0.105 | 1.439 | 0.023 | **-2.00 -1.00** | **0.00** | **1.00** |  | **2.00** |

**hydroxyguanosine decreased in PD hydroxyguanosine increased in PD**

# B Total-cholesterol

### Study name Statistics for each study Hedges's g and 95% CI

|  | **Hedges's g** | **Lower limit** | **Upper limit** | **p-Value** |  |  |  | |
| --- | --- | --- | --- | --- | --- | --- | --- | --- |
| Kirbas et al. 2014 | -0.773 | -1.217 | -0.328 | 0.001 |  |  |  |  |
| Gregório et al. 2013 | 0.317 | 0.057 | 0.577 | 0.017 |  |  |  |  |
| Kim et al. 2017 | -0.148 | -0.480 | 0.183 | 0.380 |  |  |  |  |
| Du et al. 2012 | -0.159 | -0.633 | 0.314 | 0.510 |  |  |  |  |
| Guo et al. 2015 | -0.555 | -0.675 | -0.435 | 0.000 |  |  |  |  |
| Huang et al. 2007 | -0.221 | -0.476 | 0.034 | 0.090 |  |  |  |  |
| Andican et al. 2012 | 0.423 | -0.066 | 0.912 | 0.090 |  |  |  |  |
| Watfa et al. 2011 | -0.127 | -0.782 | 0.527 | 0.703 |  |  |  |  |
| Zhang et al. 2017 | -0.346 | -0.658 | -0.033 | 0.030 |  |  |  |  |
| Ikeda et al. 2011 | -0.592 | -0.850 | -0.334 | 0.000 |  |  |  |  |
| Duran et al. 2011 | -0.289 | -0.612 | 0.035 | 0.080 |  |  |  |  |
|  | -0.241 | -0.455 | -0.026 | 0.028 |  |  |  |  |
|  |  |  |  |  | **-2.00 -1.00** |  | **0.00 1.00** | **2.00** |

**total-cholesterol decreased in PD total-cholesterol increased in PD**

Forest plot for random-effects meta-analysis on differences in blood hydroxyguanosine and total-cholesterol concentrations between Parkinson's Disease (PD) patients and healthy cognitive control (HC) . The sizes of the squares are proportional to study weight. CI, confidence interval.

**Glutathione**

**eFigure 3**

**A**

### Study name Statistics for each study Hedges's g and 95% CI

|  | **Hedges's g** | **Lower limit** | **Upper limit** | **p-Value** |  | |
| --- | --- | --- | --- | --- | --- | --- |
| Gruden et al. 2012 | -0.546 | -1.066 | -0.026 | 0.040 |  |  |
| Gironi et al. 2011 | 0.000 | -0.496 | 0.496 | 1.000 |  |  |
| Yuan et al. 2016 | -1.347 | -1.780 | -0.915 | 0.000 |  |  |
| Baillet et al. 2010 | 0.011 | -0.518 | 0.540 | 0.967 |  |  |
| Cristalli et al. 2012 | -0.729 | -1.006 | -0.452 | 0.000 |  |  |
| Watfa et al. 2011 | 0.074 | -0.580 | 0.729 | 0.824 |  |  |
| Kumudini et al. 2014 | -0.884 | -1.114 | -0.655 | 0.000 |  |  |
|  | -0.535 | -0.884 | -0.185 | 0.003 |  |  |
|  |  |  |  |  | **-2.00 -1.00 0.00 1.00** | **2.00** |

**glutathione decreased in PD glutathione increased in PD**

# B TBARS

**Study name Statistics for each study Hedges's g and 95% CI**

|  | **Hedges's g** | **Lower limit** | **Upper limit** | **p-Value** |  | | | | | |
| --- | --- | --- | --- | --- | --- | --- | --- | --- | --- | --- |
| Chen et al. 2009 | 0.667 | 0.446 | 0.888 | 0.000 |  |  |  |  |  |  |
| Baillet et al. 2010 | 1.600 | 0.991 | 2.209 | 0.000 |  |  |  |  |  |  |
| Gironi et al. 2011 | 0.000 | -0.496 | 0.496 | 1.000 |  |  |  |  |  |  |
| Ahlskog et al. 1995 | 0.038 | -0.541 | 0.618 | 0.896 |  |  |  |  |  |  |
| Kalra et al. 1992 | 0.836 | 0.263 | 1.410 | 0.004 |  |  |  |  |  |  |
| Molina et al. 1992 | -0.033 | -0.484 | 0.418 | 0.886 |  |  |  |  |  |  |
| Kumudini et al. 2014 | 1.436 | 1.190 | 1.682 | 0.000 |  |  |  |  |  |  |
| Gokce Cokal et al. 2017 | -0.068 | -0.564 | 0.428 | 0.788 |  |  |  |  |  |  |
| Naduthota et al. 2017 | 1.989 | 1.591 | 2.387 | 0.000 |  |  |  |  |  |  |
| de Farias et al. 2016 | 1.807 | 1.370 | 2.245 | 0.000 |  |  |  |  |  |  |
|  | 0.836 | 0.367 | 1.305 | 0.000 |  |  |  |  |  |  |
|  |  |  |  |  | **-4.00** | **-2.00** | **0.00** |  | **2.00** | **4.00** |

**TBARS decreased in PD TBARS increased in PD**

# eFgure 3

**C**

**Nitrite**

**nitrites decreased in PD** **nitrites increased in PD**

Forest plot for random-effects meta-analysis on differences in blood glutathione, nitrite and TBARS(thiobarbituric acid-reactive substances) concentrations between Parkinson's Disease (PD) patients and healthy cognitive control (HC) . The sizes of the squares are proportional to study weight. CI, confidence interval.

**eFigure 4**

**Sub-group analysis stratified by source of sampling for TBARS**

### Group by sample source

**Study name Statistics for each study Hedges's g and 95% CI Hedges's Lower Upper**

| plasma | Chen et al. 2009 | **g**  0.667 | **limit**  0.446 | **limit**  0.888 | **p-Value**  0.000 |  | | | | |
| --- | --- | --- | --- | --- | --- | --- | --- | --- | --- | --- |
| plasma | Baillet et al. 2010 | 1.600 | 0.991 | 2.209 | 0.000 |  |  |  |  |  |
| plasma | Kumudini et al. 2014 | 1.436 | 1.190 | 1.682 | 0.000 |  |  |  |  |  |
| plasma | de Farias et al. 2016 | 1.807 | 1.370 | 2.245 | 0.000 |  |  |  |  |  |
| plasma |  | 1.351 | 0.793 | 1.908 | 0.000 |  |  |  |  |  |
| serum | Gironi et al. 2011 | 0.000 | -0.496 | 0.496 | 1.000 |  |  |  |  |  |
| serum | Ahlskog et al. 1995 | 0.038 | -0.541 | 0.618 | 0.896 |  |  |  |  |  |
| serum | Kalra et al. 1992 | 0.836 | 0.263 | 1.410 | 0.004 |  |  |  |  |  |
| serum | Molina et al. 1992 | -0.033 | -0.484 | 0.418 | 0.886 |  |  |  |  |  |
| serum | Gokce Cokal et al. 2017 | -0.068 | -0.564 | 0.428 | 0.788 |  |  |  |  |  |
| serum | Naduthota et al. 2017 | 1.989 | 1.591 | 2.387 | 0.000 |  |  |  |  |  |
| serum |  | 0.467 | -0.289 | 1.223 | 0.226 |  |  |  |  |  |
| Overall |  | 1.039 | 0.591 | 1.488 | 0.000 |  |  |  |  |  |
|  |  |  |  |  |  | **-2.00** | **-1.00** | **0.00** | **1.00** | **2.00** |

**TBARS decreased in PD TBARS increased in PD**

Forrest plot showing pooled results comparing blood TBARS(thiobarbituric acid-reactive substances) levels between PD patients and HC subjects stratified by source of sampling (plasma and serum). The sizes of the squares are proportional to study weights.

**eFigure 5**

# Sub-group analysis stratified by source of sampling for total-cholesterol

**Group by** **sample source**

**Study name Statistics for each** **study Hedges's g and 95% CI Hedges's Lower Upper**

| plasma | Gregório et al. 2013 | **g**  0.317 | **limit**  0.057 | **limit**  0.577 | **p-Value**  0.017 |  | | | | | | |
| --- | --- | --- | --- | --- | --- | --- | --- | --- | --- | --- | --- | --- |
| plasma | Andican et al. 2012 | 0.423 | -0.066 | 0.912 | 0.090 |  |  |  |  |  |  |  |
| plasma | Watfa et al. 2011 | -0.127 | -0.782 | 0.527 | 0.703 |  |  |  |  |  |  |  |
| plasma | Zhang et al. 2017 | -0.346 | -0.658 | -0.033 | 0.030 |  |  |  |  |  |  |  |
| plasma | Duran et al. 2011 | -0.289 | -0.612 | 0.035 | 0.080 |  |  |  |  |  |  |  |
| plasma |  | -0.009 | -0.347 | 0.329 | 0.959 |  |  |  |  |  |  |  |
| serum | Kirbas et al. 2014 | -0.773 | -1.217 | -0.328 | 0.001 |  |  |  |  |  |  |  |
| serum | Kim et al. 2017 | -0.148 | -0.480 | 0.183 | 0.380 |  |  |  |  |  |  |  |
| serum | Du et al. 2012 | -0.159 | -0.633 | 0.314 | 0.510 |  |  |  |  |  |  |  |
| serum | Guo et al. 2015 | -0.555 | -0.675 | -0.435 | 0.000 |  |  |  |  |  |  |  |
| serum | Huang et al. 2007 | -0.221 | -0.476 | 0.034 | 0.090 |  |  |  |  |  |  |  |
| serum | Ikeda et al. 2011 | -0.592 | -0.850 | -0.334 | 0.000 |  |  |  |  |  |  |  |
| serum |  | -0.421 | -0.606 | -0.236 | 0.000 |  |  |  |  |  |  |  |
| Overall |  | -0.326 | -0.489 | -0.164 | 0.000 |  |  |  |  |  |  |  |
|  |  |  |  |  |  | **-2.00** | **-1.00** |  | **0.00** |  | **1.00** | **2.00** |

**total-cholesterol decreased in PD total-cholesterol increased in PD**

Forrest plot showing pooled results comparing blood total-cholesterol levels between PD patients and HC subjects stratified by source of sampling (plasma and serum). The sizes of the squares are proportional to study weights.

**A**

**eFigure 6**

**2.00**

**1.79**

**1.58**

**1.37**

**Hedges's g**

**1.16**

**0.95**

**0.74**

**0.53**

**0.32**

**0.11**

**-0.10**

## Regression of age on Hedges's g

**Meta-regression for TBARS**

**2.00**

**1.79**

**1.58**

**1.37**

**Hedges's g**

**1.16**

**0.95**

**0.74**

**0.53**

**0.32**

**0.11**

**-0.10**

## Regression of gender on Hedges's g

**49.33 51.81 54.30 56.78 59.27 61.75 64.23 66.72 69.20 71.69 74.17**

**age**

**44.97 48.85 52.73 56.61 60.49 64.38 68.26 72.14 76.02 79.90 83.79**

**gender**

**2.00**

**1.79**

**1.58**

**1.37**

**Hedges's g**

**1.16**

**0.95**

**0.74**

**0.53**

**0.32**

**0.11**

**-0.10**

## Regression of publication year on Hedges's g

**1989.50 1992.50 1995.50 1998.50 2001.50 2004.50 2007.50 2010.50 2013.50 2016.50 2019.50**

**publication year**

# B

**eFigure 6**

**Meta-regression for total-cholesterol**

**0.60**

**0.46**

**0.32**

**0.18**

**Hedges's g**

**0.04**

**-0.10**

**-0.24**

**-0.38**

**-0.52**

**-0.66**

**-0.80**

### Regression of age on Hedges's g

**0.60**

**0.46**

**0.32**

**0.18**

**Hedges's g**

**0.04**

**-0.10**

**-0.24**

**-0.38**

**-0.52**

**-0.66**

**-0.80**

**Regression of gender on Hedges's g**

**57.48 59.66 61.85 64.03 66.22 68.40 70.58 72.77 74.95 77.14 79.32**

**age**

**30.26 33.94 37.62 41.30 44.98 48.67 52.35 56.03 59.71 63.39 67.07**

**gender**

**0.60**

**0.46**

**0.32**

**0.18**

**Hedges's g**

**0.04**

**-0.10**

**-0.24**

**-0.38**

**-0.52**

**-0.66**

**-0.80**

## Regression of publication year on Hedges's g

**2006.00 2007.20 2008.40 2009.60 2010.80 2012.00 2013.20 2014.40 2015.60 2016.80 2018.00**

**publication year**

# eFigure 6 C

**0.00**

**-0.10**

**-0.20**

**-0.30**

**Hedges's g**

**-0.40**

**-0.50**

**-0.60**

**-0.70**

**-0.80**

**-0.90**

**-1.00**

### Regression of age on Hedges's g

**Meta-regression for uric acid**

**0.00**

**-0.10**

**-0.20**

**-0.30**

**Hedges's g**

**-0.40**

**-0.50**

**-0.60**

**-0.70**

**-0.80**

**-0.90**

**-1.00**

### Regression of gender on Hedges's g

**48.76 51.45 54.14 56.82 59.51 62.20 64.89 67.58 70.26 72.95 75.64**

**age**

**30.25 34.32 38.39 42.45 46.52 50.59 54.66 58.73 62.79 66.86 70.93**

**gender**

**0.00**

**-0.10**

**-0.20**

**-0.30**

**Hedges's g**

**-0.40**

**-0.50**

**-0.60**

**-0.70**

**-0.80**

**-0.90**

**-1.00**

### Regression of publication year on Hedges's g

**2006.00 2007.20 2008.40 2009.60 2010.80 2012.00 2013.20 2014.40 2015.60 2016.80 2018.00**

**publication year**

- 1. Association between age , gender ,publication year and effective size (Hedges’s g) for TBARS. **(B)** Association between age ,gender ,publication year and effective size (Hedges’s g) for total-cholesterol. **(C)** Association between age ,gender ,publication year and effective size (Hedges’s g) for uric acid. The sizes of the circles are proportional to study weights.

**eReference**

1. Abe T, Isobe C, Murata T, Sato C, Tohgi H. Alteration of 8-hydroxyguanosine concentrations in the cerebrospinal fluid and serum from patients with Parkinson's disease. *Neuroscience Letters.* 2003;336(2):105-108.

2. Ahlskog JE, Uitti RJ, Low PA, et al. No evidence for systemic oxidant stress in Parkinson's or Alzheimer's disease. *Mov Disord.* 1995;10(5):566-573.

3. Ahmed SS, Santosh W. Metallomic profiling and linkage map analysis of early Parkinson's disease: a new insight to aluminum marker for the possible diagnosis. *PLoS One.* 2010;5(6):e11252.

4. Alimonti A, Ristori G, Giubilei F, et al. Serum chemical elements and oxidative status in Alzheimer's disease, Parkinson disease and multiple sclerosis. *Neurotoxicology.* 2007;28(3):450-456.

5. Andican G, Konukoglu D, Bozluolcay M, Bayulkem K, Firtiina S, Burcak G. Plasma oxidative and inflammatory markers in patients with idiopathic Parkinson's disease. *Acta Neurol Belg.* 2012;112(2):155-159.

6. Andreadou E, Nikolaou C, Gournaras F, et al. Serum uric acid levels in patients with Parkinson's disease: their relationship to treatment and disease duration. *Clin Neurol Neurosurg.* 2009;111(9):724-728.

7. Annanmaki T, Muuronen A, Murros K. Low plasma uric acid level in Parkinson's disease. *Mov Disord.* 2007;22(8):1133-1137.

8. Annanmaki T, Pohja M, Parviainen T, Hakkinen P, Murros K. Uric acid and cognition in Parkinson's disease: a follow-up study. *Parkinsonism Relat Disord.* 2011;17(5):333-337.

9. Arnal N, Cristalli DO, de Alaniz MJ, Marra CA. Clinical utility of copper, ceruloplasmin, and metallothionein plasma determinations in human neurodegenerative patients and their first-degree relatives. *Brain Res.* 2010;1319:118-130.

10. Baillet A, Chanteperdrix V, Trocme C, Casez P, Garrel C, Besson G. The role of oxidative stress in amyotrophic lateral sclerosis and Parkinson's disease. *Neurochemical research.* 2010;35(10):1530-1537.

11. Bharucha KJ, Friedman JK, Vincent AS, Ross ED. Lower serum ceruloplasmin levels correlate with younger age of onset in Parkinson's disease. *J Neurol.* 2008;255(12):1957-1962.

12. Bogdanov M, Matson WR, Wang L, et al. Metabolomic profiling to develop blood biomarkers for Parkinson's disease. *Brain : a journal of neurology.* 2008;131(Pt 2):389-396.

13. Boll MC, Sotelo J, Otero E, Alcaraz-Zubeldia M, Rios C. Reduced ferroxidase activity in the cerebrospinal fluid from patients with Parkinson's disease. *Neurosci Lett.* 1999;265(3):155-158.

14. Bostantjopoulou S, Katsarou Z, Frangia T, et al. Endothelial function markers in parkinsonian patients with hyperhomocysteinemia. *J Clin Neurosci.* 2005;12(6):669-672.

15. Brewer GJ, Kanzer SH, Zimmerman EA, et al. Subclinical zinc deficiency in Alzheimer's disease and Parkinson's disease. *Am J Alzheimers Dis Other Demen.* 2010;25(7):572-575.

16. Cabrera-Valdivia F, Jimenez-Jimenez FJ, Molina JA, et al. Peripheral iron metabolism in patients with Parkinson's disease. *J Neurol Sci.* 1994;125(1):82-86.

17. Chen CM, Liu JL, Wu YR, et al. Increased oxidative damage in peripheral blood correlates with severity of Parkinson's disease. *Neurobiology of disease.* 2009;33(3):429-435.

18. Constantinescu R, Andreasson U, Holmberg B, Zetterberg H. Serum and cerebrospinal fluid urate levels in synucleinopathies versus tauopathies. *Acta Neurol Scand.* 2013;127(2):e8-12.

19. Cristalli DO, Arnal N, Marra FA, de Alaniz MJ, Marra CA. Peripheral markers in neurodegenerative patients and their first-degree relatives. *J Neurol Sci.* 2012;314(1-2):48-56.

20. Cubukcu HC, Yurtdas M, Durak ZE, et al. Oxidative and nitrosative stress in serum of patients with Parkinson's disease. *Neurol Sci.* 2016;37(11):1793-1798.

21. de Farias CC, Maes M, Bonifacio KL, et al. Highly specific changes in antioxidant levels and lipid peroxidation in Parkinson's disease and its progression: Disease and staging biomarkers and new drug targets. *Neurosci Lett.* 2016;617:66-71.

22. Du G, Lewis MM, Shaffer ML, et al. Serum cholesterol and nigrostriatal R2* values in Parkinson's disease. *PLoS One.* 2012;7(4):e35397.

23. Duran R, Barrero FJ, Morales B, Luna JD, Ramirez M, Vives F. Oxidative stress and aminopeptidases in Parkinson's disease patients with and without treatment. *Neurodegener Dis.* 2011;8(3):109-116.

24. Forte G, Alimonti A, Pino A, et al. Metals and oxidative stress in patients with Parkinson's disease. *Ann Ist Super Sanita.* 2005;41(2):189-195.

25. Forte G, Bocca B, Senofonte O, et al. Trace and major elements in whole blood, serum, cerebrospinal fluid and urine of patients with Parkinson's disease. *J Neural Transm (Vienna).* 2004;111(8):1031-1040.

26. Fukushima T, Tan X, Luo Y, Kanda H. Serum vitamins and heavy metals in blood and urine, and the correlations among them in Parkinson's disease patients in China. *Neuroepidemiology.* 2011;36(4):240-244.

27. Gangania MK, Batra J, Kushwaha S, Agarwal R. Role of Iron and Copper in the Pathogenesis of Parkinson's Disease. *Indian J Clin Biochem.* 2017;32(3):353-356.

28. Gatto EM, Carreras MC, Pargament GA, et al. Neutrophil function, nitric oxide, and blood oxidative stress in Parkinson's disease. *Mov Disord.* 1996;11(3):261-267.

29. Gazzaniga GC, Ferraro B, Camerlingo M, Casto L, Viscardi M, Mamoli A. A case control study of CSF copper, iron and manganese in Parkinson disease. *Ital J Neurol Sci.* 1992;13(3):239-243.

30. Gironi M, Bianchi A, Russo A, et al. Oxidative imbalance in different neurodegenerative diseases with memory impairment. *Neurodegener Dis.* 2011;8(3):129-137.

31. Gokce Cokal B, Yurtdas M, Keskin Guler S, et al. Serum glutathione peroxidase, xanthine oxidase, and superoxide dismutase activities and malondialdehyde levels in patients with Parkinson's disease. *Neurol Sci.* 2017;38(3):425-431.

32. Gonzalez-Aramburu I, Sanchez-Juan P, Jesus S, et al. Genetic variability related to serum uric acid concentration and risk of Parkinson's disease. *Mov Disord.* 2013;28(12):1737-1740.

33. Grau AJ, Willig V, Fogel W, Werle E. Assessment of plasma lactoferrin in Parkinson's disease. *Mov Disord.* 2001;16(1):131-134.

34. Gregório ML, Pinhel MAS, Sado CL, et al. Impact of Genetic Variants of Apolipoprotein E on Lipid Profile in Patients with Parkinson's Disease. *BioMed Research International.* 2013;2013:1-7.

35. Gruden MA, Yanamandra K, Kucheryanu VG, et al. Correlation between protective immunity to alpha-synuclein aggregates, oxidative stress and inflammation. *Neuroimmunomodulation.* 2012;19(6):334-342.

36. Guo X, Song W, Chen K, et al. The serum lipid profile of Parkinson's disease patients: a study from China. *Int J Neurosci.* 2015;125(11):838-844.

37. Haglin L, Backman L. Covariation between plasma phosphate and daytime cortisol in early Parkinson's disease. *Brain Behav.* 2016;6(12):e00556.

38. Hemmati-Dinarvand M, Taher-Aghdam AA, Mota A, Zununi Vahed S, Samadi N. Dysregulation of serum NADPH oxidase1 and ferritin levels provides insights into diagnosis of Parkinson's disease. *Clin Biochem.* 2017;50(18):1087-1092.

39. Hozumi I, Hasegawa T, Honda A, et al. Patterns of levels of biological metals in CSF differ among neurodegenerative diseases. *J Neurol Sci.* 2011;303(1-2):95-99.

40. Huang X, Chen H, Miller WC, et al. Lower low-density lipoprotein cholesterol levels are associated with Parkinson's disease. *Mov Disord.* 2007;22(3):377-381.

41. Ikeda K, Nakamura Y, Kiyozuka T, et al. Serological profiles of urate, paraoxonase-1, ferritin and lipid in Parkinson's disease: changes linked to disease progression. *Neurodegener Dis.* 2011;8(4):252-258.

42. Jesus S, Perez I, Caceres-Redondo MT, et al. Low serum uric acid concentration in Parkinson's disease in southern Spain. *Eur J Neurol.* 2013;20(1):208-210.

43. Jimenez-Jimenez FJ, Fernandez-Calle P, Martinez-Vanaclocha M, et al. Serum levels of zinc and copper in patients with Parkinson's disease. *J Neurol Sci.* 1992;112(1-2):30-33.

44. Jimenez-Jimenez FJ, Molina JA, Aguilar MV, et al. Cerebrospinal fluid levels of transition metals in patients with Parkinson's disease. *J Neural Transm (Vienna).* 1998;105(4-5):497-505.

45. Kalra J, Rajput AH, Mantha SV, Chaudhary AK, Prasad K. Oxygen free radical producing activity of polymorphonuclear leukocytes in patients with Parkinson's disease. *Mol Cell Biochem.* 1992;112(2):181-186.

46. Kalra J, Rajput AH, Mantha SV, Prasad K. Serum antioxidant enzyme activity in Parkinson's disease. *Mol Cell Biochem.* 1992;110(2):165-168.

47. Kikuchi A, Takeda A, Onodera H, et al. Systemic increase of oxidative nucleic acid damage in Parkinson's disease and multiple system atrophy. *Neurobiology of disease.* 2002;9(2):244-248.

48. Kim JH, Hwang J, Shim E, Chung EJ, Jang SH, Koh SB. Association of serum carotenoid, retinol, and tocopherol concentrations with the progression of Parkinson's Disease. *Nutr Res Pract.* 2017;11(2):114-120.

49. Kim TH, Lee JH. Serum uric acid and nigral iron deposition in Parkinson's disease: a pilot study. *PLoS One.* 2014;9(11):e112512.

50. Kirbas A, Kirbas S, Cure MC, Tufekci A. Paraoxonase and arylesterase activity and total oxidative/anti-oxidative status in patients with idiopathic Parkinson's disease. *J Clin Neurosci.* 2014;21(3):451-455.

51. Kouti L, Noroozian M, Akhondzadeh S, et al. Nitric oxide and peroxynitrite serum levels in Parkinson's disease: correlation of oxidative stress and the severity of the disease. *Eur Rev Med Pharmacol Sci.* 2013;17(7):964-970.

52. Kumudini N, Uma A, Naushad SM, Mridula R, Borgohain R, Kutala VK. Sexual dimorphism in xenobiotic genetic variants-mediated risk for Parkinson's disease. *Neurol Sci.* 2014;35(6):897-903.

53. Loeffler DA, Klaver AC, Coffey MP, Aasly JO, LeWitt PA. Increased Oxidative Stress Markers in Cerebrospinal Fluid from Healthy Subjects with Parkinson's Disease-Associated LRRK2 Gene Mutations. *Front Aging Neurosci.* 2017;9:89.

54. Lolekha P, Wongwan P, Kulkantrakorn K. Association between serum uric acid and motor subtypes of Parkinson's disease. *J Clin Neurosci.* 2015;22(8):1264-1267.

55. Medeiros MS, Schumacher-Schuh A, Cardoso AM, et al. Iron and Oxidative Stress in Parkinson's Disease: An Observational Study of Injury Biomarkers. *PLoS One.* 2016;11(1):e0146129.

56. Miao J, Liu J, Xiao L, et al. The Single Nucleotide Polymorphism rs1014290 of the SLC2A9 Gene Is Associated with Uric Acid Metabolism in Parkinson's Disease. *Parkinsons Dis.* 2017;2017:7184927.

57. Molina JA, Jimenez-Jimenez FJ, Fernandez-Calle P, et al. Serum lipid peroxides in patients with Parkinson's disease. *Neurosci Lett.* 1992;136(2):137-140.

58. Naduthota RM, Bharath RD, Jhunjhunwala K, et al. Imaging biomarker correlates with oxidative stress in Parkinson's disease. *Neurol India.* 2017;65(2):263-268.

59. Nicoletti G, Crescibene L, Scornaienchi M, et al. Plasma levels of vitamin E in Parkinson's disease. *Arch Gerontol Geriatr.* 2001;33(1):7-12.

60. Paraskevas GP, Kapaki E, Petropoulou O, Anagnostouli M, Vagenas V, Papageorgiou C. Plasma levels of antioxidant vitamins C and E are decreased in vascular parkinsonism. *Journal of the Neurological Sciences.* 2003;215(1-2):51-55.

61. Qin XL, Zhang QS, Sun L, Hao MW, Hu ZT. Lower Serum Bilirubin and Uric Acid Concentrations in Patients with Parkinson's Disease in China. *Cell Biochem Biophys.* 2015;72(1):49-56.

62. Sakuta H, Suzuki K, Miyamoto T, et al. Serum uric acid levels in Parkinson's disease and related disorders. *Brain Behav.* 2017;7(1):e00598.

63. Sampat R, Young S, Rosen A, et al. Potential mechanisms for low uric acid in Parkinson disease. *J Neural Transm (Vienna).* 2016;123(4):365-370.

64. Serra JA, Dominguez RO, de Lustig ES, et al. Parkinson's disease is associated with oxidative stress: comparison of peripheral antioxidant profiles in living Parkinson's, Alzheimer's and vascular dementia patients. *J Neural Transm (Vienna).* 2001;108(10):1135-1148.

65. Shukla R, Rajani M, Srivastava N, Barthwal MK, Dikshit M. Nitrite and malondialdehyde content in cerebrospinal fluid of patients with Parkinson's disease. *Int J Neurosci.* 2006;116(12):1391-1402.

66. Sterling NW, Lichtenstein M, Lee EY, et al. Higher Plasma LDL-Cholesterol is Associated with Preserved Executive and Fine Motor Functions in Parkinson's Disease. *Aging Dis.* 2016;7(3):237-245.

67. Sun CC, Luo FF, Wei L, et al. Association of serum uric acid levels with the progression of Parkinson's disease in Chinese patients. *Chin Med J (Engl).* 2012;125(4):583-587.

68. Urakami K, Sano K, Matsushima E, et al. Decreased superoxide dismutase activity in erythrocyte in Parkinson's disease. *Jpn J Psychiatry Neurol.* 1992;46(4):933-936.

69. van Kamp GJ, Mulder K, Kuiper M, Wolters EC. Changed transferrin sialylation in Parkinson's disease. *Clin Chim Acta.* 1995;235(2):159-167.

70. Vieru E, Koksal A, Mutluay B, Dirican AC, Altunkaynak Y, Baybas S. The relation of serum uric acid levels with L-Dopa treatment and progression in patients with Parkinson's disease. *Neurol Sci.* 2016;37(5):743-747.

71. Vinish M, Anand A, Prabhakar S. Altered oxidative stress levels in Indian Parkinson's disease patients with PARK2 mutations. *Acta Biochim Pol.* 2011;58(2):165-169.

72. Wang F, Yu SY, Zuo LJ, et al. Excessive Iron and alpha-Synuclein Oligomer in Brain are Relevant to Pure Apathy in Parkinson Disease. *J Geriatr Psychiatry Neurol.* 2016;29(4):187-194.

73. Wang L, Hu W, Wang J, et al. Impact of serum uric acid, albumin and their interaction on Parkinson's disease. *Neurol Sci.* 2017;38(2):331-336.

74. Watfa G, Dragonas C, Brosche T, et al. Study of telomere length and different markers of oxidative stress in patients with Parkinson's disease. *J Nutr Health Aging.* 2011;15(4):277-281.

75. Younes-Mhenni S, Aissi M, Mokni N, et al. Serum copper, zinc and selenium levels in Tunisian patients with Parkinson's disease. *Tunis Med.* 2013;91(6):402-405.

76. Yuan Y, Tong Q, Zhang L, et al. Plasma antioxidant status and motor features in de novo Chinese Parkinson's disease patients. *Int J Neurosci.* 2016;126(7):641-646.

77. Zhang HN, Guo JF, He D, et al. Lower serum UA levels in Parkinson's disease patients in the Chinese population. *Neurosci Lett.* 2012;514(2):152-155.

78. Zhang L, Wang X, Wang M, et al. Circulating Cholesterol Levels May Link to the Factors Influencing Parkinson's Risk. *Front Neurol.* 2017;8:501.

79. Zhao N, Jin L, Fei G, Zheng Z, Zhong C. Serum microRNA-133b is associated with low ceruloplasmin levels in Parkinson's disease. *Parkinsonism Relat Disord.* 2014;20(11):1177-1180.

80. Zuo LJ, Yu SY, Hu Y, et al. Serotonergic dysfunctions and abnormal iron metabolism: Relevant to mental fatigue of Parkinson disease. *Scientific reports.* 2016;6(1):19.
